# Complement C1 Esterase Inhibitor Levels Linked to Infections and Contaminated Heparin-Associated Adverse Events

**DOI:** 10.1371/journal.pone.0034978

**Published:** 2012-04-13

**Authors:** Zhao-Hua Zhou, Trina Chen, Kamalpreet Arora, Kenneth Hyams, Steven Kozlowski

**Affiliations:** 1 Division of Monoclonal Antibodies, Office of Pharmaceutical Sciences, Center for Drug Evaluation and Research, Food and Drug Administration, Silver Spring, Maryland, United States of America; 2 Office of Biological Products, Office of Pharmaceutical Sciences, Center for Drug Evaluation and Research, Food and Drug Administration, Silver Spring, Maryland, United States of America; Drexel University College of Medicine, United States of America

## Abstract

Activation of kinin-kallikrein and complement pathways by oversulfated-chondroitin-sulfate (OSCS) has been linked with recent heparin-associated adverse clinical events. Given the fact that the majority of patients who received contaminated heparin did not experience an adverse event, it is of particular importance to determine the circumstances that increase the risk of a clinical reaction. In this study, we demonstrated by both the addition and affinity depletion of C1inh from normal human plasma, that the level of C1inh in the plasma has a great impact on the OSCS-induced kallikrein activity and its kinetics. OSCS-induced kallikrein activity was dramatically increased after C1inh was depleted, while the addition of C1inh completely attenuated kallikrein activity. In addition, actual clinical infection can lead to increased C1inh levels. Plasma from patients with sepsis had higher average levels of functional C1inh and decreased OSCS-induced kallikrein activity. Lastly, descriptive data on adverse event reports suggest cases likely to be associated with contaminated heparin are inversely correlated with infection. Our data suggest that low C1inh levels can be a risk factor and high levels can be protective. The identification of risk factors for contact system-mediated adverse events may allow for patient screening and clinical development of prophylaxis and treatments.

## Introduction

All medications have the potential to produce adverse events (AEs) [1] and such adverse events lead to significant morbidity and mortality [2], [3]. Between late 2007 and early 2008 there was an increase in heparin-associated AEs. According to the Centers for Disease Control and Prevention (CDC) and the Food and Drug Administration (FDA), these AEs resembled anaphylaxis and occurred in hundreds of patients. Associated symptoms and signs included hypotension, facial swelling, tachycardia, urticaria, nausea and in some cases death [4], [5], [6], [7], [8]. A “heparin-like” contaminant, oversulfated chondroitin sulfate (OSCS), found in up to 30% of suspect lots of heparin, was associated with the AEs [5], [6]. Despite the likely distribution of millions of contaminated heparin doses [4] only hundreds of adverse events were reported [8]. Even with potential under-reporting, this suggests the majority of patients who received the same lots of contaminated heparin did not experience an adverse event. Thus, exposure to OSCS required additional co-factors or patient susceptibilities to cause clinical reactions.

The major symptoms of this cluster of heparin associated AEs are characteristic of anaphylaxis [6]. Anaphylaxis includes immunologic (e.g., IgE-mediated or immune complex-mediated) and non-immunologic mechanisms (e.g., mediated through other activators of mast cell degranulation) that cause clinically indistinguishable reactions [9]. Signs and symptoms of anaphylaxis vary, but cutaneous features (urticaria, angioedema, and erythema) and decreased blood pressure are the most common overall [9]. Although the AEs associated with contaminated heparin often included hypotension, gastrointestinal symptoms were also common and urticaria was relatively rare [4], [8]. Thus, IgE-mediated allergic reactions or mast cell degranulation were unlikely explanations for the heparin-associated adverse events [10]. IgG-mediated hypersensitivity reactions were also unlikely explanations due to the rapid onset of the AEs [10]. However OSCS activated the contact system enzyme kallikrein leading to amidolytic activity *in vitro*, and OSCS caused a hypotensive response in pigs [6].

While this study of the contact system provided a potential biologic link between the contaminant and the contact system-associated anaphylactoid reactions seen in affected patients, some animals injected with OSCS did not develop any clinical signs [6]. This is not unexpected as the majority of patients that received contaminated lots of heparin did not develop any clinical symptoms. Apparently, OSCS alone does not necessarily lead to development of symptoms. Since the contact system is a major target of OSCS and a likely cause of the clinical responses, susceptibility factors could include components and/or regulators of the contact system.

We hypothesized that the C1 esterase inhibitor (C1inh) level is a key factor in determining the outcome of OSCS exposure. This is based upon several facts. First, C1inh, an alpha-2-globulin and member of the serpin family of serine protease inhibitors, is a major inhibitor for both the complement classical pathway and contact system [11], [12], [13]. While C1inh is the only inhibitor of C1r and C1s, the classical complement pathway proteases [14], [15], it also regulates intrinsic coagulation via inactivation of factor XIa [16], [17]. Importantly in our context, C1inh regulates kinin generation via inactivation of factors XIIa and plasma kallikrein. Second, many of the reported OSCS-contaminated heparin AE reactions were similar to reactions caused by C1inh-deficiency, i.e., hereditary angioedema (HAE) [12], [18], [19]. In addition to the inherited C1inh-deficiency, an acquired C1inh-deficiency can also occur due to consumption of C1inh in the situations of a sudden or continuous activation of the complement pathways leading to a transient or prolonged decrease in C1inh levels [11], [18], [20], [21]. Acquired C1inh deficiency is often associated with lymphoproliferative disease and/or C1inh reactive autoantibodies [11]. C1inh levels may also vary in response to a variety of situations, such as infection, inflammation and autoimmune diseases (e.g.,systemic lupus erythematosus or SLE) [22], [23]. Third, knocking out the C1 inhibitor gene to generate homozygous- and heterozygous- C1inh deficient mice, revealed increased vascular permeability and angioedema mediated by bradykinin via the bradykinin B_2_ receptor (Bk2r) [19].

If C1inh is a key factor that contributed to the OSCS linked adverse events, then we would predict that higher or lower levels of C1inh in the plasma should have an enhancing or inhibitory impact, respectively, on the OSCS-induced contact system activation. In this study, several types of analyses were used to test this hypothesis.

## Methods

### Materials

OSCS-contaminated heparin lots and un-contaminated heparin lots were obtained by the FDA from Baxter Healthcare (1000 U/ml or 5000 U/ml in 10 ml and 30 ml vials) [7]. Synthetic oversulfated chondroitin sulfate (OSCS) was obtained from the Division of Pharmaceutical Analysis, FDA. St. Louis, MO. Chondroitin sulfate A (CSA) was purchased from Sigma (St. Louis, MO). Purified human complement C1 esterase inhibitor (C1inh) was purchased from Sigma. Normal human plasma and purified human prekallikrein was purchased from Innovative Research (Novi, Michigan). Purified human Factor XIIa and Factor XII deficient plasma supplied by Hyphen BioMed (France) was purchased through Innovative Research. Recombinant human complement components C1r and C1s were purchased from R&D Systems (Minneapolis, MN). Anti-complement component C1 inhibitor mouse monoclonal antibody was obtained from the AntibodyShop (Denmark), purified peroxidase-conjugated goat anti-human C1inh IgG was purchased from Cedarlane laboratories (Canada). Protein A/G-Sepharose was purchased from BioVision (Mountain View, CA). PE anti-human complement C3 monoclonal antibody was purchased from Cedarlane laboratories.

### Human Plasma Samples

Human plasma samples from patients and normal individuals were obtained from Bioreclamation, Inc. (Hicksville, NY). All the human samples were de-identified in such a manner that subjects could not be identified, directly or through identifiers linked to the subjects. Other than the clinical descriptor (e.g. sepsis), only age and gender of the person were collected and recorded (FDA RIHSC Protocol #10-054D).

### Affinity Depletion of C1inh from Human Plasma

16 sterile micro tubes (Sarstedt Inc., Newton, NC) each with 0.5 ml Protein A/G Sepharose beads were washed with cold PBS twice and centrifuged at 10,000 g. To half of the tubes (designated as “Anti-C1inh beads”) 150 µg monoclonal mouse anti-human C1inh IgG and 1 mg of goat anti-human C1inh polyclonal IgG were added while to the other half of the tubes (designated as “Control beads”) either same amount of PBS or irrelevant goat IgG was added. The tubes were incubated with rotation at 4°C for 20 minutes followed by three washes with cold PBS. Then 1 ml of normal human plasma and of Factor XII-deficient human plasma were added to the “Anti-C1inh beads” or “Control beads” tubes. The tubes were mixed by rotation for 20 minutes at 4°C, followed by centrifugation at 10,000 g. The bead-treated plasma samples were then transferred to a second tube with the same beads and the steps were again repeated for a total of four rounds resulting in control human plasma, C1inh-reduced plasma, Factor XII-deficient plasma and C1inh−/FXII- double deficient plasma. The plasma was then aliquoted and frozen at −80°C. C1inh levels in the plasma were determined by ELISA and more than 98% of C1inh antigen was removed by this method. The above described plasma treatment did not induce “cold activation” and kallikrein level was within base line as determined by s-2302.

### Kallikrein Amidolytic Activity Assay

Normal human plasma, C1inh-deficient plasma, factor XII-deficient plasma or C1inh−/FXII double deficient human plasma were treated with various concentration of OSCS, OSCS-contaminated heparin, uncontaminated heparin, and chondroitin sulfate A for 10 minutes at 37°C. The samples were then diluted 1∶10 with 50 mM Tris-HCl and an equal amount of s-2302 chromogenic substrate (H-D-Pro-Phe-Arg-p-nitroaniline [pNA]·2HCl, DiaPharma Group, Inc., West Chester, Ohio) was added. The samples were then incubated at 37°C for 10 minutes, followed by the addition of 20% acetic acid to stop the reaction and the spectrophotometric optical density (OD) was determined at 405 nm. It is worthy to mention that s-2302 is also substrate for FXa, FXIa, FXIIa and thrombin, but assay conditions as described above can make the substrate highly specific for kallikrein (100% reactivity for plasma kallikrein vs. <3∼10% for other factors).

### Bacteria Treatment of Plasma and C1inh Deposition

Gram-negative *E.* coli *BL21* bacteria were cultured in Luria-Bertani (LB) broth, until an OD_600_ of 0.3 was reached. Then the bacteria were washed thoroughly with cold PBS and 2×10^8^ bacteria were incubated with 50 µg of monoclonal polyreactive IgM [24] at room temperature for 30 minutes and washed with cold PBS. Then the antibody coated bacteria were added to 100 µl of normal human plasma for 5 minutes at 37°C followed by centrifugation at 10,000 g for 2 minutes. The plasma was then removed and diluted with PBS and the C1inh levels were tested by ELISA. To evaluate the C1inh deposited on bacteria, the bacteria were washed twice with cold PBS, followed by the addition of goat anti-human C1inh IgG-peroxidase, incubation at 4°C for 30 minutes, four washes with cold PBS, centrifugation and the addition of 1 ml ABST substrate. Samples were then incubated at 37°C for 20 minutes, centrifuged again, and the supernatants were read at 405 nm for OD. Bacteria that had not been treated with plasma were used as a negative control. The bacterial treatment did not generate kallikrein activity as determined by assay with the substrate s-2302.

### OSCS-Induced Kallikrein Kinetics and Dose Response

Normal and C1inh-deficient human plasma that had been treated or not treated with polyreactive antibody were mixed with various concentrations of OSCS as indicated. After incubation for 10 minutes at 37°C, samples were diluted with 50 mM PH 7.8 Tris-HCl and s-2302 substrate was added with continued incubation and shaking for 10 minutes at 37°C, followed by the addition of 20% acetic acid to stop the reaction. The OD at 405 nm was measured to determine the kallikrein activity. For OSCS-induced kallikrein kinetics, 25 µg/ml of OSCS was added to normal plasma and C1inh-deficient plasma at different time points. Then s-2302 was added and incubated at 37°C for 10 minutes, immediately followed by the addition of 20% acetic acid to stop the reaction. Sample OD at 405 nm was measured.

### Competition for C1inh by C1r, C1s and FXIIa

To optimize the dose of C1inh for the inhibition of FXIIa, different doses of C1inh (from 2.5 ng to 1000 ng) were incubated with 1 ng of FXIIa in 20 µl of 50 mM Tris-HCl buffer at 37°C for 5 minutes, then added into 1 µg purified prekallikrein (in 10 µl Tris-HCl) and the incubation continued at 37°C for 1 minute. Then 20 µl Tris-HCl and 50 µl s-2302 were added and incubated at 37°C for 10 minutes followed by the addition of 50 µl acetic acid to stop reaction. Kallikrein activity was evaluated by reading the OD at 405 nm. For the competition assay, 1 µg of prekallikrein was added with 0.5 µg of C1inh, a mixture of 0.5 µg C1inh with 0.5 µg C1s and 0.5 µg C1r, or 50 µl Tris-HCl buffer, and incubated at 37°C for 5 minutes. Then 1 ng of FXIIa in 10 µl Tris-HCl buffer was added and the samples incubated at 37°C for another 5 minutes. 50 µl s-2302 was then added and the samples were incubated at 37°C for 10 minutes. The reaction was stopped by the addition of 50 µl 20% acetic acid and the OD at 405 nm was measured.

### Functional C1inh in plasma

Functional C1inh was determined by a chromogenic assay using the Technochrom C1-inh kit (DiaPharma Group, Inc., West Chester, Ohio) according to the manufacture's instruction with some modifications. Briefly, all the reagents provided in the kit were reconstituted and prepared; all the plasma samples and reference standard were diluted with Sample Buffer A at a ratio of 1∶11 and put on ice. 50 µl diluted plasma samples and standard reference were added into 96-well polystyrene plates with duplication and kept on ice. Then 25 µl/well C1s was added, followed by incubation at 37°C for 5 minutes. Plates were then again put on ice, 125 µl of substrate C1-1 was then added to each well and the plates were incubated at 37°C for 3 minutes. Plates were placed on ice and 50 µl/well of 50% acetic acid was added immediately. The OD at 405 nm was read on a Perkin Elmer Victor Multilabel Plate Reader (Waltham, Massachusetts). Functional C1inh levels were calculated using the standard reference curve.

### Statistical Analysis

Duplicate samples were evaluated and experiments were repeated at least three times except as noted. Data are presented as mean or mean ± standard error of the mean. For patient samples, data are expressed as mean of duplicates for each sample and mean ± SD for the group. The Pearson product-moment correlation coefficient was used to evaluate the correlation between variables. Difference between groups and all paired comparisons were subjected to unpaired or two-tailed Student's t tests, respectively. Significance was set at a p value of less than 0.05. All differences noted in experiments with multiple paired comparisons were significant at a p value of <0.01.

### Adverse Event Report Descriptions

Adverse event reports submitted to the FDA were evaluated using Empirica Signal 7.2 software. Queries to count cases used CBAERS BestRep (S+C) data. This data set contains the best representative cases, includes suspect as well as concomitant drugs and has duplicate removal. All queries were limited to cases where the active ingredient heparin was reported as a suspect or concomitant drug and the term “dialysis” was present in the narrative. This set of case reports was divided into two groups. One group had event dates from 11/1/2007 to 3/31/2008, the timeframe of the OSCS contamination. The second contained all the other reports from 1/1/2000 to 12/31/2010 as a control group. Additional queries were then limited by other reported events or medications. Event terms used in these queries were Medical Dictionary for Regulatory Activities (MedDRA) Preferred Terms (PT) or High Level Group Terms (HGLT) terms and are described in Appendix S1. Drug terms used in the queries are terms based on the Anatomical Therapeutic Chemical (ATC) classification system and also described in Appendix S1.

## Results

### C1 inhibitor regulates OSCS-induced kallikrein activity

**Figure 1:**
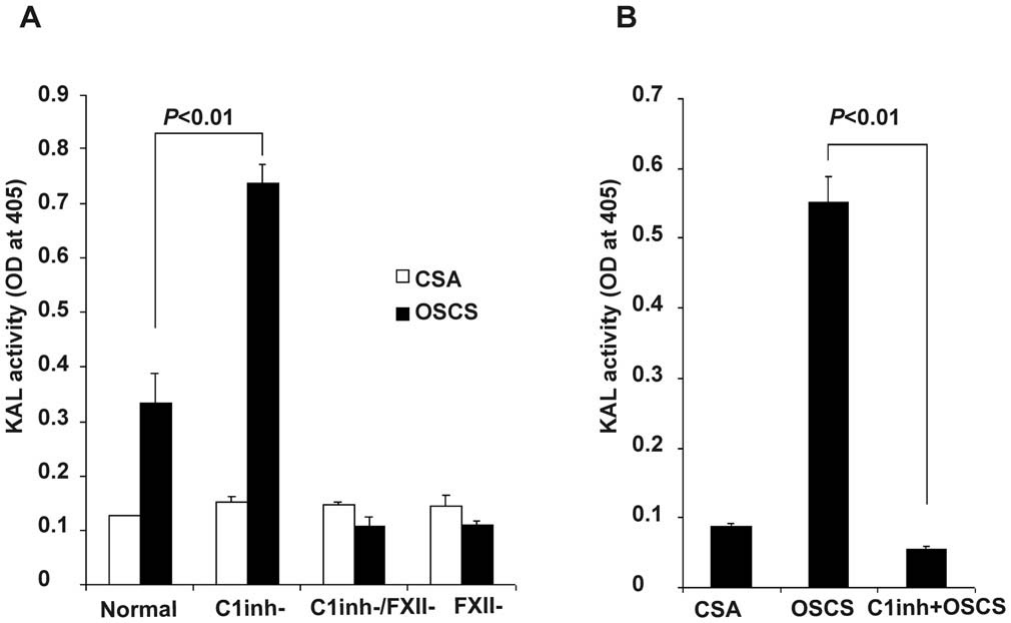
C1 inhibitor regulates OSCS-induced kallikrein activity. (A) Normal human plasma, plasma depleted of C1 inhibitor, plasma depleted of factor XII, and plasma depleted of both C1 inhibitor and factor XII, were incubated with chondroitin sulfate A (CSA) or over sulfated chondroitin sulfate A (OSCS). The effect of kallikrein amidolytic activity was assessed by the addition of the s-2302 chromogenic substrate. (B) OSCS-induced kallikrein activity was totally inhibited by addition of 200 µg/ml of C1 inhibitor. At least three independent experiments were completed with duplicate samples for the assay.

To determine if C1inh plays any role in OSCS-induced kallikrein amidolytic activity, normal human plasma and the same plasma depleted of C1inh were incubated with OSCS and then kallikrein activity was compared using a chromogenic assay. As shown in figure 1a, OSCS induced kallikrein activity in normal plasma and when C1inh was depleted, kallikrein activity was dramatically increased. Kallikrein activity by OSCS in both normal and C1inh-deficient plasma was FXII-dependent, since the depletion of FXII alone or both FXII and C1inh together led to an absence of kallikrein activity. A control glycosaminoglycan (GAG), chondroitin sulfate A (CSA) did not induce kallikrein activity independent of C1inh or FXII levels (Figure 1a). The data also indicate the experimental depletion of FXII and C1inh did not induce additional kallikrein activity in the controls. The inhibitory role of C1inh in the OSCS-induced kallikrein activity was confirmed by adding highly purified C1inh into C1inh-depleted plasma before the addition of OSCS. Kallikrein activity was totally inhibited with the addition of 200 µg/ml of C1inh (Figure 1b).

### C1 inhibitor can be depleted by complement activation leading to increased OSCS-induced kallikrein activity

**Figure 2:**
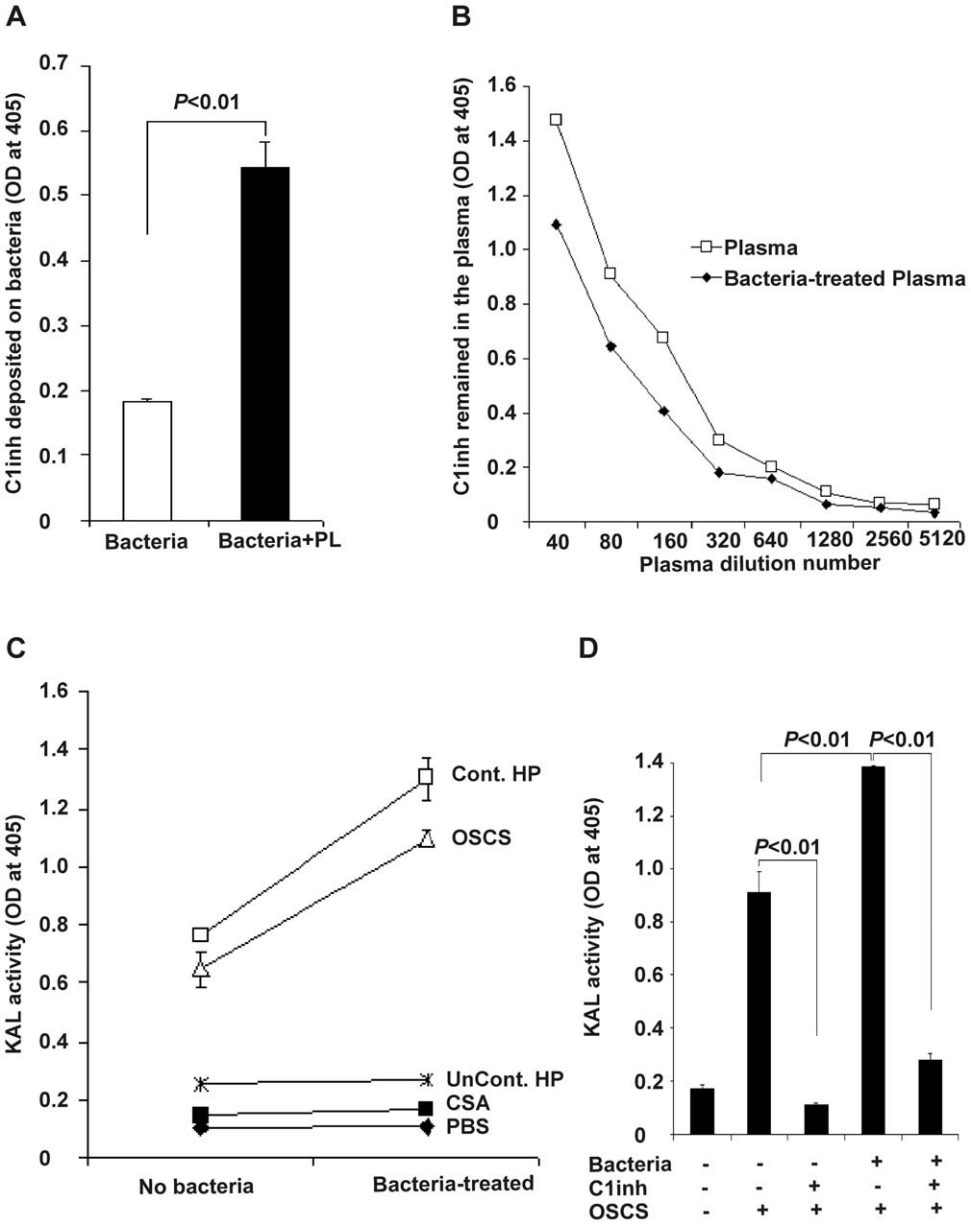
Bacteria incubation consumes C1 inhibitor and increases OSCS-induced kallikrein activity. (A) Natural antibody-coated *E. coli* BL21 bacteria were incubated with human plasma at 37°C, washed by PBS, and the level of C1 inhibitor deposited on the bacteria was determined by addition of horseradish peroxidase-conjugated anti-C1 inhibitor antibody and then developement with ABTS substrate as described in the Materials and Methods. (B) An ELISA assay showed decreased C1 inhibitor levels in plasma treated with antibody-coated *E. coli* BL21 bacteria. (C) OSCS and OSCS-contaminated heparin induced kallikrein activity were increased in plasma treated with antibody-coated bacteria as compared to untreated plasma. PBS, CSA or uncontaminated heparin did not induce kallikrein activity in either plasma. (D) Kallikrein activity in plasma treated with or without antibody-coated *E. coli* BL21 bacteria, C1 inhibitor (200 µg/ml), or OSCS (25 µg/ml) was measured. Data shown were representative of three independent experiments.

To rule out an effect on kallikrein activity specific to affinity depletion of C1inh, we evaluated the effect of C1inh consumption through a biologically relevant pathway, bacterial activation of complement [24]. Complement C1 and C3 fixation on bacteria were detected by immunostaining and FACS analysis (data not shown), and there was significant deposition of C1inh on bacterial surfaces after incubation with plasma (Figure 2a). As expected from the observed C1inh deposition, the plasma C1inh level was decreased in bacteria-treated plasma (Figure 2b). While the bacterial treatment alone did not generate kallikrein activity, OSCS-induced kallikrein activity was strikingly increased in the bacteria-treated plasma as compared to non-bacteria treated control plasma (Figure 2c). Kallikrein activity induced by OSCS contaminated-heparin has a similar pattern as compared with the kallikrein activity induced by synthetic OSCS. A PBS control, CSA and uncontaminated-heparin did not induce kallikrein activity in either bacteria-treated or untreated plasma (Figure 2c). The addition of C1inh completely inhibited the OSCS-induced kallikrein activity in both normal plasma and bacteria-treated plasma (Figure 2d).

### Kinetic and dose response of OSCS-induced kallikrein activity

**Figure 3:**
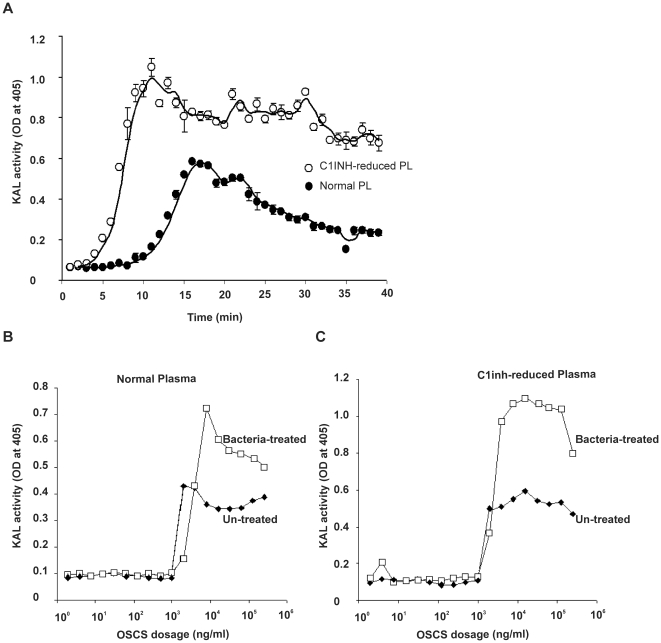
OSCS kallikrein activation kinetics and dose response. (A) Kinetics of kallikrein activation in normal plasma and C1inh-depleted plasma with the addition of 25 µg/ml of OSCS. Kallikrein activity was also determined in (B) normal human plasma and (C) C1inh-depleted plasma with different doses of OSCS. The OSCS dose response was also evaluated with plasma samples treated with antibody-coated bacteria. Experiments were repeated independently twice. Duplicate samples were used in panel A.

A kinetic study showed that OSCS-induced kallikrein activity reached a peak within 10 minutes in C1inh-depleted plasma while the OSCS-induced kallikrein activity took 15 minutes to peak in normal plasma (Figure 3a). Kallikrein activity was reduced to half-maximal levels in approximately 10 minutes in normal plasma while a similar reduction took more than 30 minutes in C1inh-reduced plasma. Thus, the kinetics as well as the levels of kallikrein activity were impacted by C1inh depletion. The more rapid induction, higher levels and longer duration of kallikrein activity observed in the C1inh-depleted plasma suggest an earlier, higher and longer exposure to kallikrein products, such as bradykinin. Bradykinin (BK) or related molecules are considered likely mediators of the OSCS contaminated heparin-induced clinical symptoms, such as hypotension and gastrointestinal symptoms [25].

The dose response of OSCS was similar in both normal plasma and C1inh-depleted plasma with increases in kallikrein activity occurring at levels above 1 µg/ml; however, the magnitude of kallikrein activity was higher in C1inh-depleted plasma (Figure 3b and 3c).

After a robust increase, kallikrein activity remained relatively flat within a broad range (5 to 150 µg/ml) of OSCS concentrations. This finding is consistent with the observation of the CDC study [4] that no major difference in symptoms was noted between lots above and below 20% contaminant. Of note, a prior study using AERS data [8] indicated that a lot with a low concentration of OSCS (∼3%) was less likely to be associated with a time-to-event of 10 minutes or less in selected cases.

The addition of antibody coated bacteria to plasma increased the magnitude of the OSCS induced kallikrein activity in both normal (Figure 3b) and C1inh depleted plasma (Figure 3c). The increased magnitude of OSCS induced kallikrein activity in C1inh depleted plasma may be due to further depletion of C1inh through complement activation although other bacterial effects are possible.

### Complement components C1r and C1s compete with FXIIa for C1inh interaction

**Figure 4:**
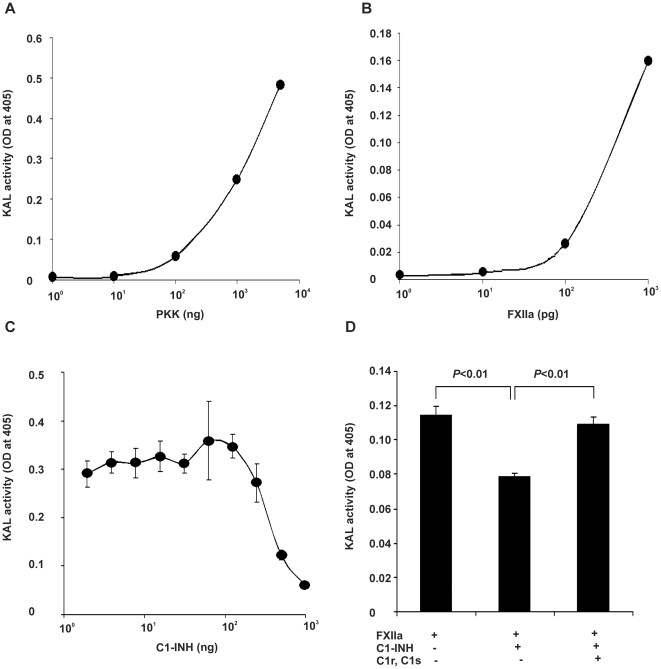
Complement components C1r and C1s compete with FXIIa for C1inh. Kallikrein activity of (A) different doses of prekallikrein incubated with a fixed dose (1 ng) of FXIIa; (B) a fixed dose (0.5 µg) of prekallikrein incubated with different doses of FXIIa; (C) different doses of C1inh premixed with a fixed dose (1 ng) of FXIIa before combining with prekallikrein (1 µg). (D) Kallikrein activity of 0.5 µg of prekallikrein incubated with different combinations of FXIIa, C1inh and C1r/C1s. Experiments were repeated independently at least three times. Duplicate samples were used in panels C and D.

To confirm the role of C1inh consumption in the above experiments, a simplified model with purified proteins was used to determine if the interaction of complement components C1r and C1s with C1inh will have any impact on the contact system component, activated FXII (FXIIa). A mixture of purified plasma prekallikrein and FXIIa generate kallikrein as shown in figure 4. The kallikrein activity was dependent on the level of prekallikrein (Figure 4a) and FXIIa (Figure 4b). Pre-incubation of C1inh with FXIIa has a dose-dependent inhibition on the FXIIa activation of prekallikrein into kallikrein (Figure 4c). This inhibition of FXIIa by C1inh was blocked by the addition of C1r and C1s to C1inh (Figure 4d). This finding supports complement consumption of C1inh as the mechanism for the increased susceptibility of bacteria-treated plasma to OSCS induced kallikrein activation.

### Plasma of patients with higher C1inh levels exhibited lower OSCS-induced kallikrein activity

**Figure 5:**
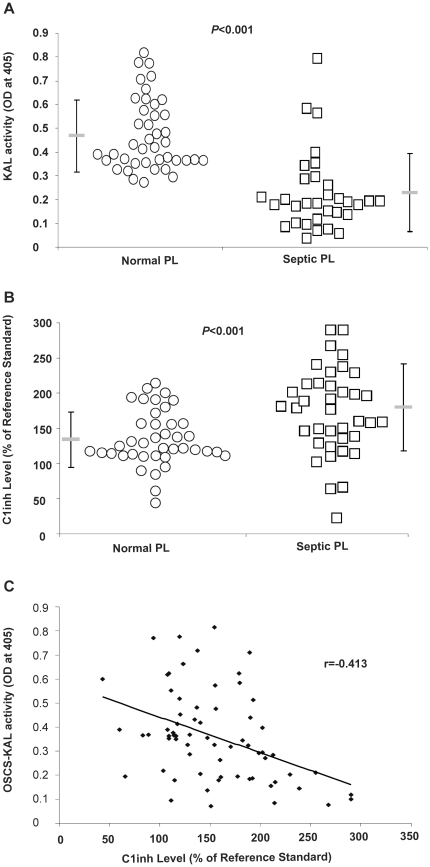
OSCS-induced kallikrein activation in patient plasma samples. (A) OSCS-induced kallikrein activation and (B) functional C1inh levels in plasma samples from normal individuals (n = 40) and from patients with septic shock (n = 32). (C) The relationship of OSCS-induced kallikrein activity and functional C1inh levels using the same samples. The error bars correspond to mean ± SD for each patient group. The correlation coefficient r = −0.413 indicates a negative association between functional C1inh levels and OSCS-induced kallikrein activation. Data shown here were representative of three independent experiments.

To check if there is any relationship between OSCS-induced contact system activation and the level of C1 inhibitor in patient populations, OSCS was added to plasma samples from normal individuals and patients with sepsis. Although C1 inhibitor levels can be depleted due to consumption by bacterial activation of complement *in vitro*, *in vivo* C1inh levels can increase due to elevated C1inh synthesis in response to infections [26]. As shown in figure 5a, OSCS-induced kallikrein activity was significantly lower in plasma from septic patients than in plasma of normal individuals. This suggested an overall increase in C1inh levels in sepsis and in some studies on sepsis, C1-inh levels were increased depending on the clinical state of the patients [27], [28], [29]. C1 inh levels were evaluated in our samples by measuring C1 inh function rather than C1 inh antigen since functional C1 inh is more relevant for adverse events [23]. We also observed a significant difference of the average functional C1inh level between normal plasma samples and septic patient samples. The average normal plasma C1inh level was 130% of the reference standard and the average septic plasma C1inh level was 175% of the reference standard. The large diversity of the functional C1inh level within samples of both groups indicates C1inh level varies among individuals and depends on the pathophysiological status of patients. C1inh levels in septic patients was as low as <20% or as high as 300% of the reference standard (Figure 5b).

For sepsis, this variability may depend on sample timing with depletion of C1inh in very early infection followed by a rapid increase with upregulated C1inh synthesis. If the patient deteriorates C1 inh levels may again decrease. Nujiens et al demonstrated that, despite elevated antigenic levels of C1 inh, some patients with sepsis have increased levels of inactivated C1 inh and relative functional C1inh deficiency [23]. This suggests further evaluation of inactivated C1 inh in prognosis and C1 inh in treatment of deteriorating patients. However a number of studies besides our own have observed increased functional C1 inh activity in subgroups of septic patients [27], [28], [29]

Since the differences in functional C1inh levels across the sample populations did not fully explain the pattern of OSCS-induced kallikrein activity observed, we evaluated the relationship between OSCS-induced kallikrein activity and functional C1inh in each sample. This evaluation demonstrated a significant negative relationship between C1inh functional activity and OSCS-induced kallikrein activity with an R of −0.413 (Figure 5c). Although the data showed a lower functional C1inh index is generally related to a higher OSCS-induced kallikrein activity in the plasma, the R value and outlier points suggest there are other potential factors, such as Factor XII. Levels of Factor XII and prekallikrein have been shown to decrease in some septic patients [27], [29]. However, these results suggest the level of C1inh is likely to be an important factor in patient susceptibility to OSCS and there may be enough variability in C1inh levels to impact the clinical outcome of OSCS exposure.

### A lower frequency of adverse events reports was associated with infection during the OSCS contamination timeframe

Although in rare patients sepsis was associated with low levels of functional C1 inhibitor, in general C1inh levels in the septic plasma were higher, and OSCS-induced kallikrein activity was lower in the septic patient samples. To look for a negative correlation between infections and OSCS-mediated adverse events, we compared adverse event reports during the time period of heparin contamination with other adverse event reports (Table 1) as described in the Materials and Methods. There was a very large increase in rate of adverse event reports during the timeframe of OSCS contamination. As expected, the frequency of events associated with the contaminant, hypotension and gastrointestinal symptoms, more than doubled during this period. Although the overall rate of reporting infection and sepsis was not that different between the groups, when the groups were filtered for either hypotension or gastrointestinal symptoms, signs associated with contaminated heparin exposure, infections and sepsis were less frequent during the timeframe of OSCS contamination. Of note, for cases reporting gastrointestinal symptoms, sepsis was less than half as frequent during the OSCS contamination timeframe. Another measure of infection was the use of anti-infective medications. Use of anti-infectives was far less likely to be reported with adverse events during the OSCS contamination timeframe. This decreased frequency of reporting was also observed when cases were filtered for either hypotension or gastrointestinal symptoms and when a slightly different set of anti-infective search terms were used. The relative differences in frequency between infections and anti-infectives may relate to the severity and nature of the infections. Those relative differences decrease when the data is filtered for hypotension or gastrointestinal symptoms.

**Table 1:** Characteristics of AE associated with Heparin & Dialysis 2000–2010.

	All (No Filter)		Hypotension		GI symptoms	
Characteristic	All except 11/2007–3/2008	11/2007–3/2008	All except 11/2007–3/2008	11/2007–3/2008	All except 11/2007–3/2008	11/2007–3/2008
AE Case Reports						
All	1585	817	327	381	308	426
Rate/month	12.5	163.4	2.6	76.2	2.4	85.2
Contact System Sx						
Hypotension	20.6%	46.6%				
Gastrointestinal Sx	19.4%	52.1%				
Infections						
Infections	28.7%	32.4%	48.3%	39.6%	48.4%	32.2%
Sepsis-Septic	13.8%	14.9%	29.7%	24.1%	28.9%	13.8%
BK Metabolism						
ACE inhib.	22.5%	21.5%	24.2%	25.7%	25.6%	27.5%
Anti-infectives						
Antiinfectives	44.5%	21.7%	39.8%	27.6%	37.3%	20.0%
Antibact./Antibiot.	41.1%	20.8%				
Control Drugs						
ATii antagon.	9.7%	8.2%				
Statins	25.4%	24.8%				
Diabetes Meds	29.9%	25.9%				
Control Events						
HIT	5.9%	6.5%				
Myopathy-Myolysis	2.8%	2.9%				

Since the mechanism that links elevated kallikrein activity to the observed symptoms had been postulated to be the generation of kinins, such as BK [25], any factor that increases the levels or prolongs the duration of BK might impact the risk of OSCS exposure. ACE inhibitors (ACEi) can increase the levels and prolong the duration of BK by decreasing BK degradation [25]. To evaluate this, we compared the frequency of reported ACEi use during the period of OSCS contamination to the control period. No increase in ACEi use was noted during the time period of the OSCS contamination. Only very small differences in reported ACEi use were observed if the data were filtered for either hypotension or gastrointestinal symptoms.

As passive adverse event reporting has many limitations, there are alternative explanations for any unobserved or observed differences in frequencies. To better understand the limits of these observations, a number of control medications and events were also evaluated. The frequency of reports with angiotensin II antagonists, drugs with some mechanistic similarity to ACEi also did not notably change during the time period of OSCS contamination. There was also no notable change in the reporting of statin use but there was a small decrease in the reporting of medications for diabetes. Reports of heparin induced thrombocytopenia and a variety of myopathies did not have a notable change in frequency during the period of OSCS contamination. This finding makes it less likely that the observed decreases in reporting of infections and anti-infective use during the time period of OSCS contamination are due to dilution of reported clinical problems.

## Discussion

The adverse events associated with OSCS contaminated heparin have been linked to kallikrein activation. Although OSCS-driven increases in anaphylactic toxins C3a and C5a have also been demonstrated in vitro, OSCS-driven kallikrein activation has been shown in both in vitro and in vivo studies [6]. Pigs treated with OSCS also developed hypotension, a predominant feature of the adverse events seen in OSCS exposed patients. Factor XIIa was also shown to be important in kallikrein activation by OSCS [6]. OSCS was additionally shown to lead to bradykinin generation [25]. Bradykinin is a 9-amino acid proinflammatory peptide that can mediate pain, potent vasodilation and increased vascular permeability, [25], [30] leading to accumulation of fluid in the interstitium. These biological effects can cause clinical symptoms, including gastrointestinal symptoms such as nausea, vomiting, diarrhea, abdominal pain and lower blood pressure [8], [10], [25], [30], [31]. This supports a role for the contact system in contaminated heparin adverse events.

Of note, in the Kishimoto study [6] not all the exposed animals exposed to OSCS or contaminated heparin developed hypotension. This is not surprising since the vast majority of patients who received contaminated heparin lots did not report adverse events [4], [8]. It is important to understand what factors may have altered patient susceptibility to OSCS and factors that influence levels of BK would be likely candidates.

**Figure 6:**
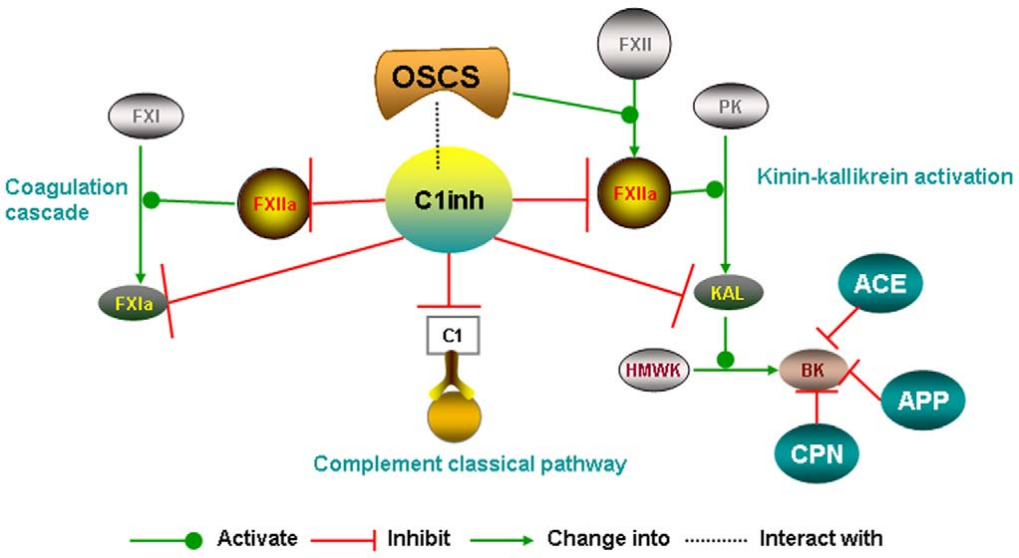
Factors that can influence bradykinin levels. The level of bradykinin (BK) is determined by the levels of kallikrein (KAL), high molecular weight kininogen (HMWK) and enzymes that degrade BK, such a angiotensin converting enzyme (ACE), aminopeptidase P (APP) and neutral carboxypeptidase (CPN). Activated factor XII (FXIIa) generates KAL from prekallikrein (PK). C1 inhibitor (C1inh) can inhibit both FXIIa and KAL. OSCS can facilitate FXIIa activity through enhancing its generation from FXII and/or stabilizing a complex of FXIIa, PK and HMWK. OSCS may also have a direct effect on C1inh. Other factors in the complement pathway and coagulation cascade can interact with C1inh and thus decrease C1inh availability for limiting BK production.

Factors that can influence BK levels are diagramed in figure 6; all of the listed factors may play a role in susceptibility to OSCS. OSCS can facilitate FXIIa conversion of pre-kallikrein (PK) to kallikrein, which is a pivotal factor in contact system activation [10], [31]. OSCS has been shown to bind FXIIa as well as FXII and may enhance conversion of FXII to FXIIa as well as stabilize the complex of FXIIa and PK [31]. This increases FXIIa conversion of pre-kallikrein into kallikrein and kallikrein can then proteolytically cleave high molecular-weight kininogen (HMWK) into bradykinin (BK). C1 inhibitor has been shown to be the major inhibitor of FXIIa and an important inhibitor of kallikrein [32]. The critical role of C1 inhibitor in blocking two key mediators of BK generation suggests that it is an important factor in OSCS-mediated adverse events. OSCS may also have a direct effect on C1inh, as other glycosaminoglycans have been demonstrated to bind C1inh and impact C1inh functionality [14]. Our results support a key role of C1 inhibitor in OSCS associated adverse events. We found that C1inh regulates the OSCS-induced FXII-dependent kallikrein activity. OSCS-induced kallikrein activity was dramatically increased after depletion of C1inh from plasma. The addition of C1inh completely attenuated OSCS-induced kallikrein activity. Plasma from patients with septic shock patients had a higher average C1inh level and exhibited lower average OSCS-induced kallekrein activity. Plasma from patients with lower levels of C1inh had higher OSCS-induced kallikrein activity.

The CDC study [4] suggested a high frequency of drug allergy (mostly antibiotics) in patients with OSCS-mediated adverse events. We only observed a small increase in OSCS-mediated kallikrein activation in those samples as compared to normal plasma (data not shown)

To better understand the relationship between adverse events, infection and thus C1inh, we evaluated adverse event reports associated with heparin and dialysis. Reports of the expected symptoms of OSCS-contaminated heparin were less likely to be associated with infection and sepsis during the time period of OSCS contaminated heparin. In addition, reports of anti-infective use were less frequent during the time period of OSCS-contaminated heparin. The lower use of antiinfectives was even more striking than the lower rates on infection. This may indicate that more serious infections were even less likely to be associated with OSCS-mediated adverse events. However, the possibility that specific antibiotics were protective independent of infection was considered. Although unlikely, the high rate of antibiotic allergies observed by the CDC study [4] could reflect a lower likelihood of specific antibiotic use by allergic patients. Thus we evaluated whether the decreased use of antibiotics was limited to one class of drugs. Although the decreased reporting of different antibiotic classes was variable, it was seen across multiple classes making a highly specific protective effect less likely (data not shown).

The evaluation of adverse event reports has a number of limitations, as they represent passive reports with the challenges of inconsistent reporting and reporting that lacks essential details [8]. Public knowledge of a product recall, such as occurred with heparin, stimulates increased reporting of adverse events. Although a much higher rate of reporting may be seen for both related and unrelated events, the increased reporting could potentially dilute out some other adverse events. This could lead to an artificial reduction in the relative reporting of events such as infections. We had a number of controls to indicate that the reductions in frequency we observed were unlikely to be spurious. Reported control events were not reduced in frequency and although anti-infective use was reduced throughout the high reporting period, infection reports were only reduced in the context of OSCS-mediated symptoms. Thus it is unlikely dilution accounts for our observations. Adverse event reports are often duplicated so the database we used has an electronic de-duplication algorithm. Our data does not appear to differ notably in terms of the major adverse events identified from either the CDC study [4] or the manually de-duplicated database of heparin adverse event reports [8].

As shown in figure 6, when BK is generated, levels are limited due to degradation by three BK kininases, angiotensin-converting enzyme (ACE), aminopeptidase P (APP) and carboxypeptidase N (CPN), which cleave BK at the 7–8, 1–2, and 8–9 positions, respectively [32], [33], [34]. Due to these enzymes, BK is usually degraded within minutes after its generation and does not accumulate [32]. In the kinin-kallikrein system, any shift of the generation or degradation of BK can change levels of BK. A recent study by Adam et. al. showed OSCS induction of BK in ACEi treated human plasma [25]. This finding supports kallikrein generation of BK as the mechanism for OSCS induced adverse events and suggests that ACEi use could be another risk factor for OSCS mediated adverse events. The CDC study [4] noted 26% of patients with contaminated heparin mediated events were on ACEi, a prevalence that did not appear to be greater than expected. Our study did not find a meaningful increase in the frequency of cases reporting ACEi use during the time period of the OSCS contamination. Since ACEi blockade of BK degradation should lead to increased accumulation of BK, the lack of a relationship between ACEi and adverse events needs explanation. The use of ACEi may lead to compensatory increases in other mechanisms to clear BK such as APP, CPN or other proteases. ACEi associated angioedema only occurs in 0.1 to 1% of treated patients suggesting a large fraction of treated patients have compensatory mechanisms to assure BK clearance [35]. A study on ACEi angioedema suggests differences in induction of Des-Arg BK instead of BK correlate with ACEi angioedema [36]. Although compensatory mechanisms and kinin specificity may explain the lack of an association of adverse events with ACEi, further evaluation is needed.

Our observations regarding C1inh have broader implications than OSCS contamination and infection. There are many other potential causes of decreased C1inh. Although the genetic deficiency of C1inh in humans, hereditary angioedema, is a very rare life-threatening disorder [11], [23], [37], [38], there may be some asymptomatic patients who are missed, including relatives of known cases [39]. Consumption of C1inh may occur in patients with autoimmunity such as SLE [40], [41], [42], [43]. Antibodies to C1 inh have been observed in some healthy individuals as well as SLE patients [43] and such antibodies may predispose healthy individuals to contact system stressors such as OSCS. Adaptive C1inh deficiency can be observed in patients with B cell lymphoma [21], [44]. Some hormone products, such as estrogen containing oral contraceptives may impact genetic C1 inh deficiency as well as lead to a mild C1 inh deficiency in unaffected individuals [39]. C1 inh decreases during open heart surgery [22] and low C1 inh levels were correlated with capillary leak syndrome in children undergoing cardiac bypass surgery [45]. Of note ∼5% of patients with heparin associated adverse events in the CDC study [4] and 16% of heparin associated adverse events in the AERS study [8] were linked to cardiac procedures.

It is likely that many inflammatory conditions can alter C1inh levels through consumption or increased synthesis. Since these opposing responses may occur together, functional C1inh levels may be dynamic and differ over time in the same patient. Real-time monitoring of C1inh activity may be useful in understanding the dynamics of susceptibility to contact system triggers. Although C1inh levels vary between patient populations, there is significant variability within a population as seen in figure 5. This suggests that in addition to all the individuals with defined pathological states that alter C1 inh levels, there will be a subset of individuals within the normal population who are more susceptible to contact system triggers. Although we demonstrate a correlation between lower levels of kallikrein generation and increased levels of functional C1inh in patient samples, the limited correlation suggests there are other important factors in addition to C1inh levels. As the patho-physiology of sepsis or infectious disease is complicated and may change multiple factors, the OSCS-induced lower kallikrein production in septic patients might be a consequence of combination of several parameters such as lower prekallikrein and/or Factor XIIa levels [28] in addition to an increased C1inh level. In addition, in the cases of sepsis, other protease inhibitors, such as alpha-2-macroglobulin, may also decrease [27]. It will be important to better understand the role of other potential factors such as those shown in figure 6. A combination of factors may fully explain the low rate of reported events.

In conclusion, several types of data indicate that C1inh level is likely to be an important factor in patient susceptibility to OSCS-contaminated heparin. The totality of data from *in vitro* studies on C1inh effects, evaluation of clinical samples and clinical adverse event reports supports the relevance of C1 inhibitor as an important factor. Although current analytics can detect future OSCS contaminations, other contaminants and/or other products may also trigger the contact system and lead to adverse events. Understanding patient susceptibilities to contact system activation may facilitate strategies to prevent such adverse events. For example, with appropriate supportive clinical studies and regulatory approval, there may be a role for C1inh in therapy or prevention when treatments with potential contact system reactions are necessary.

## Supporting Information

Appendix S1 (DOC)
