# Supplementary material for: Complement C1 Esterase Inhibitor Levels Linked to Infections and Contaminated Heparin-Associated Adverse Events
Source: PLoS One. 2012 Apr 13;7(4):e34978. doi: 10.1371/journal.pone.0034978 (PMC3325920; doi:10.1371/journal.pone.0034978)
Supplement: Appendix S1 — (DOC) [file pone.0034978.s001.doc]

**Appendix S1**

| **Table Description** | **Term Type** | **Terms included in Query** |
| --- | --- | --- |
| Hypotension | PT | Blood pressure decreased, Blood pressure systolic decreased, Hypotension |
| Gastrointestinal Sx | HGLT | Gastrointestinal signs and symptoms |
| Infections | HGLT | Ancillary infectious topics, Bacterial infectious disorders, Central nervous system infections and inflammations, Chlamydial infectious disorders, Female reproductive tract infections and inflammations, Fungal infectious disorders, Gastrointestinal infections, Infections - pathogen class unspecified, Infections - pathogen unspecified, Infections and infestations congenital, Male reproductive tract infections and inflammations, Mycobacterial infectious disorders, Mycoplasmal infectious disorders, "Ocular infections, irritations and inflammations", Protozoal infectious disorders, Rickettsial infectious disorders, Skin and subcutaneous tissue infections and infestations, Viral infectious disorders |
| Sepsis-Septic | PT | Abdominal sepsis, Anthrax sepsis, Bacterial sepsis, Biliary sepsis, Brucella sepsis, Burkholderia cepacia complex sepsis, Campylobacter sepsis, Candida sepsis, Cerebral septic infarct, Citrobacter sepsis, Clostridium difficile sepsis, Corynebacterium sepsis, Device related sepsis, Enterobacter sepsis, Enterococcal sepsis, Escherichia sepsis, Fungal sepsis, Group B streptococcus neonatal sepsis, Haemophilus sepsis, Helicobacter sepsis, Herpes sepsis, Infantile septic granulomatosis, Klebsiella sepsis, Listeria sepsis, Meningococcal sepsis, Micrococcal sepsis, Myocarditis septic, Neutropenic sepsis, Nocardia sepsis, Pelvic sepsis, Plague sepsis, Pneumococcal sepsis, Post procedural sepsis, Postpartum sepsis, Pseudallescheria sepsis, Pseudomonal sepsis, Pulmonary sepsis, Salmonella sepsis, Sepsis, Sepsis neonatal, Sepsis pasteurella, Sepsis syndrome, Septic arthritis haemophilus, Septic arthritis neisserial, Septic arthritis staphylococcal, Septic arthritis streptobacillus, Septic arthritis streptococcal, Septic embolus, Septic encephalopathy, Septic necrosis, Septic phlebitis, Septic rash, Septic shock, Serratia sepsis, Staphylococcal sepsis, Stenotrophomonas sepsis, Streptococcal sepsis, Thrombophlebitis septic, Umbilical sepsis, Urosepsis, Wound sepsis |
| Antiinfectives | ATC level 1 | Infec |
| Antibact./Antibiot. | ATC level 2 | Antibacterials For Systemic Use, Antibiotics And Chemotherapeutics For Dermatological Use |
| ACE inhib. | ATC level 4 | Ace Inhibitors And Calcium Channel Blockers, Ace Inhibitors And Diuretics, "Ace Inhibitors, Plain" |
| ATii antagon. | ATC level 4 | "Angiotensin Ii Antagonists, Plain" |
| Statins | ATC level 4 | Hmg Coa Reductase Inhibitors, "Hmg Coa Reductase Inhibitors, Other Combinations" |
| Diabetes Meds | ATC level 2 | Drugs Used In Diabetes |
| HIT | PT | Heparin-induced thrombocytopenia, Heparin-induced thrombocytopenia test, Heparin-induced thrombocytopenia test positive |
| Myopathy-Myolysis | PT | Cardiomyopathy, Cardiomyopathy acute, Cardiomyopathy alcoholic, Cardiomyopathy neonatal, Congenital myopathy, Congestive cardiomyopathy, Cytotoxic cardiomyopathy, Diabetic cardiomyopathy, HIV cardiomyopathy, Hollow visceral myopathy, Hypertensive cardiomyopathy, Hypertrophic cardiomyopathy, Ischaemic cardiomyopathy, Metabolic myopathy, Mitochondrial encephalomyopathy, Mitochondrial myopathy, Mitochondrial myopathy acquired, Myopathy, Myopathy endocrine, Myopathy toxic, Neuromyopathy, Non-obstructive cardiomyopathy, Peripartum cardiomyopathy, Restrictive cardiomyopathy, Rhabdomyolysis, Stress cardiomyopathy, Viral cardiomyopathy |
